# Characterization of mineral coatings associated with a Pleistocene‐Holocene rock art style: The Northern Running Figures of the East Alligator River region, western Arnhem Land, Australia

**DOI:** 10.1016/j.dib.2016.12.024

**Published:** 2016-12-20

**Authors:** Penelope L. King, Ulrike Troitzsch, Tristen Jones

**Affiliations:** a Research School of Earth Sciences, The Australian National University, Canberra, ACT 2601, Australia; b Department of Archaeology and Natural History, School of Culture, History and Language, The Australian National University, Canberra, ACT 2601, Australia

**Keywords:** X-ray Diffraction, Scanning Electron Microscopy energy dispersive spectroscopy, Fourier Transform infrared spectroscopy

## Abstract

This data article contains mineralogic and chemical data from mineral coatings associated with rock art from the East Alligator River region. The coatings were collected adjacent to a rock art style known as the “Northern Running Figures” for the purposes of radiocarbon dating (doi:10.1016/j.jasrep.2016.11.016; (T. Jones, V. Levchenko, P.L. King, U. Troitzsch, D. Wesley, 2017) [1]). This contribution includes raw and processed powder X-ray Diffraction data, Scanning Electron Microscopy energy dispersive spectroscopy data, and Fourier Transform infrared spectral data.

**Specifications Table**

**Table:** 

Subject area	Archeology
More specific subject area	Rock art
Type of data	Tables and Figures
How data was acquired	1. Powder X-ray diffraction (XRD) analysis (PANalytical Empyrean powder X-ray diffractometer, Research School of Chemistry, Australian National University) 2. Scanning Electron Microscope energy dispersive spectral (SEM-EDS) analysis (Hitachi 4300SE/N field emission scanning electron microscope equipped with an Oxford INCA Energy 350 EDS system at the Centre for Advanced Microscopy, Australian National University) 3. Fourier Transform Infrared (FTIR) spectroscopic analysis – mid-infrared spectral range (Bruker Tensor 27, Research School of Earth Sciences, Australian National University)
Data format	Raw and analysed
Experimental factors	Characterization of mineralogy and chemistry of mineral coatings associated with rock art
Experimental features	Analysis of minerals, their quantities and their chemical composition
Data source location	Eastern Alligator River, western Arnhem Land, Australia
Data accessibility	The data is available with this article.

**Value of the data**

• Data presented here will be useful to other researchers as a benchmark for Powder X-ray Diffraction and Fourier Transform Infrared spectra of natural oxalate-bearing mineral coatings.
• The raw XRD data may be reanalyzed with a different set of phosphate, sulfate and oxalate standards, which may help constrain the uncertainty in the Rietveld refinement values.
• The raw FTIR data may be deconvolved using other appropriate mineral databases and the results compared with the XRD Rietveld refinement values.

## 1 Data

### 1.1 Data from X-ray diffraction with Rietveld refinement fits

The oxalate mineral, whewellite, is found in all mineral crusts sampled (13–26.4 wt.%), except RLL3-1-1 where it is not detected (Table 1, Supplementary Figs. 1 and 2, Supplementary Table 1). Samples RLL32-B-S1 to RLL32-B-S4, RLL3-1-2 and RLL3-1-3 have the same mineral assemblage: whewellite and tinsleyite, with lesser taranakite, quartz and gypsum (Table 1). Sample RLL32-B-2011 is dominated by quartz and whewellite, with a little gypsum, a 10 Å-mica and a 7.1 Å-clay. Sr-crandallite or goyazite may be present at low levels (Table 1).

### 1.2 Data from Scanning Electron Microscope analysis

Backscattered electron (BSE) imaging data (Fig. 8a and 8d in [1]) indicates that the mineral crust contains at least four intimately mixed minerals (<1 µm to ~3 µm). As shown in Table 2, SEM-EDS data from the mineral crust indicates whewellite, and Ca–Al–(Sr)–phosphate(s) –crandallite, Sr–crandallite, or crandallite mixed with apatite.

### 1.3 Data from Fourier Transform Infrared spectroscopy

**Fig. 1:**
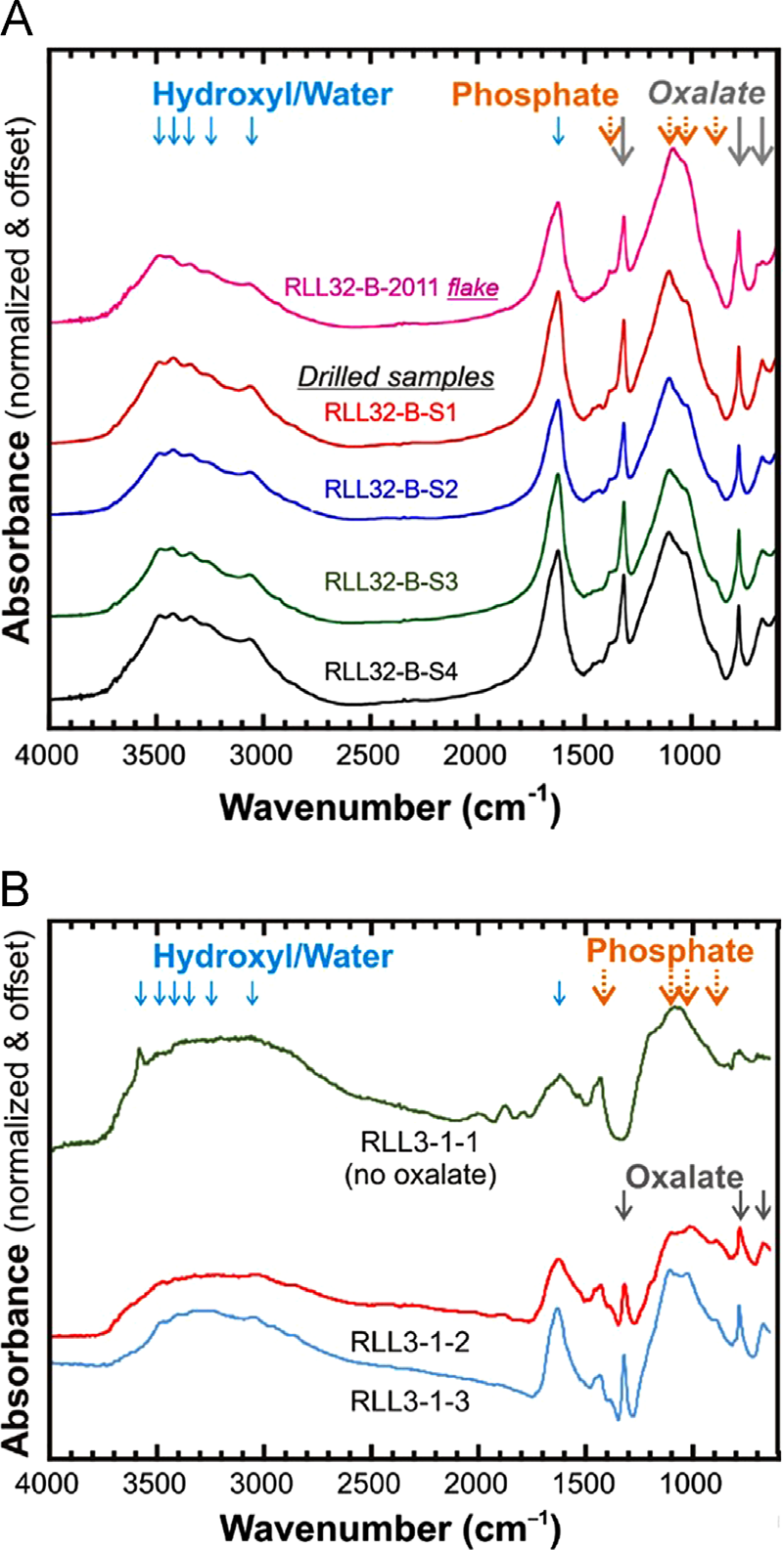
FTIR spectra of bulk samples from the mineral crusts. The positions of the bands identified in Supplementary Table 2 are indicated. (A) RLL032-B powders. (B) RLL3-1 powders. RLL3-1-1 does not contain detectable oxalate.

Fourier Transform Infrared spectra provide constraints for the presence of oxalates, sulfates, phosphates and clay minerals in the crusts (Fig. 1a and b). Infrared bands associated with the calcium oxalate (whewellite) are evident in the spectra at 1315–1320 and 780 (C_2_O_4_) and 670 cm^−1^ (water libration) and possible bands include 1430 cm^−1^ (C_2_O_4_), 3420 cm^−1^ (OH), and 1625 cm^−1^ (HOH). Phosphate minerals (crandallite, Sr-crandallite/goyazite and apatite) have bands at 1383, ~1110 and 890 cm^−1^ and 1020 cm^−1^ related to PO_4_ vibrations and 3486 cm^−1^ related to OH. Bands due to silicate minerals are found at 3246 cm^−1^ (Al_2_-OH, clay) and 1020 cm^−1^ (SiO_4_). The FTIR data does not rule out sulfate (Supplementary Table 2). Bands at 3344 and 3062 cm^−1^ are assigned to OH groups in minerals.

The six FTIR spectra obtained from the RLL032B-B site ([1], Fig. 1a) are consistent with one another with only slight differences observed in the topmost sample (RLL032-B-2011). The latter shows slightly less defined OH bands at 3490–3420 cm^−1^ and a doublet in the area near 670 cm^−1^. The FTIR data is consistent with the XRD that shows that RLL032-B-2011 differs from the rest of the samples (Fig. 1a).

RLL3-1-2 and RLL3-1-3 both contain strong oxalate bands, phosphate bands and H–O molecular species (Fig. 1b). RL3-1-1 does not show detectable oxalate, but instead contains bands between 1000–1100 cm^−1^ and 1800–2100 cm^−1^ (Fig. 1b) due to Al–O and Si–O vibrations (e.g, varsicite and quartz; Table 1, Supplementary Table 2).

## 2 Experimental design, materials and methods

### 2.1 Study area description

Mineral coatings were collected from rock walls adjacent to art described in detail by [1]. The locations are given in Table 3.

### 2.2 X-ray diffraction (XRD) methods

Samples were prepared as powders, mounted on a silicon low-background sample holder, and analyzed from 4 to 70° 2? at a spacing of 0.02626° (Supplementary Table 1). Data was collected using Bragg Brentano geometry, fixed divergence slits with *Cu Kα* radiation and a PIXcel 1D detector (active length = 3.3473°, 255 channels, 542 s per step). Minerals were identified using the SIEMENS software package Diffrac*plus* Eva 10 [2] (Supplementary Fig. 1) and quantified using Rietveld refinement [3], [4] with the program Rietica [5] (Table 1, Supplementary Fig. 2). The background was fixed manually. The weight fraction of the amorphous material *W*_*AMORPH*_ was determined for each corundum-spiked sample according to equation *W*_*AMORPH*_=1−*y*/*x,* where *y*=% corundum, and *x* is the calculated % corundum given by the program Rietica [6]. Amorphous, poorly crystallized and/or very finely grained material is identified in all samples by elevated or undulating backgrounds.

### 2.3 Scanning electron microscopy – energy dispersive spectrometry (SEM-EDS) methods

Sample RLL032-B-2011 was mounted in epoxy perpendicular to the mineral crust surface and polished to a ¼ µm diamond grit finish using kerosene, not water. SEM analysis was undertaken using a 15 kV accelerating voltage and 1 nA beam current with an approximately 2 μm beam diameter that overlapped multiple mineral phases.

### 2.4 Fourier Transform Infrared (FTIR) spectroscopy methods

Samples were ground, dried at ~100 °C, and mixed with KBr (sample:KBr = 0.6:1) and pressed into a 3 mm diameter disc held in a paper holder. Spectra were collected using a Bruker Tensor 27 with a Globar source, KBr beamsplitter and DTGS detector in transmission mode under a dry air purge from 400 to at least 4000 cm^−1^, with 4 cm^−1^ resolution and 100 scans. (Supplementary Table 3). FTIR bands were located using the OPUS software (v8.0) provided by Bruker and identified using data from the literature (Supplementary Table 2, [7], [8], [9], [10]).

## Figures and Tables

**Table 1:** Quantitative data for minerals in the crusts based on Rietveld refinement fits of X-ray diffraction data.

**Sample**	**RLL032-B-2011**	**RLL032-B-S1**	**RLL032-B-S2**	**RLL032-B-S3**	**RLL032-B-S4**	**RLL3-1-1**	**RLL3-1-2**	**RLL3-1-3**
**Scan No.**	A24950	A25292	A25302	A25301	A25290	A24950	A25292	A25302
***R***_***wp***_^a^	4.53	4.63	3.28	3.40	3.70	4.75	4.09	4.30

***Mineral- wt.% (sd)***^b^								
Amorphous material	70.5 (5.0)	53.8 (5.0)	52.3 (5.0)	65.9 (5.0)	69.5 (5.0)	28.3 (3.0)	14.9 (3.0)	40.6 (5.0)
Quartz SiO_2_	9.3 (0.7)	2.5 (0.1)	3.1 (0.1)	2.9 (0.2)	1.7 (0.2)	32.7 (0.6)	5.6 (0.3)	3.2 (0.2)
Gypsum CaSO_4._2H_2_O	1.1 (0.2)	3.7 (0.3)	3.0 (0.3)	0.8 (0.2)	1.7 (0.2)	1.8 (0.1)	3.1 (0.2)	2.0 (0.1)
Whewellite CaC_2_O_4_.H_2_O	13.0 (0.9)	20.3 (0.8)	20.1 (0.7)	17.4 (0.8)	15.1 (0.9)		26.4 (0.6)	17.4 (0.6)
Tinsleyite KAl_2_(PO_4_)_2_(OH).2H_2_O		17.7 (1.0)	18.2 (0.8)	12.9 (0.8)	11.9 (0.9)	21.5 (0.5)	48.3 (0.8)	35.2 (0.9)
Taranakite K_3_Al_5_(HPO_4_)_6_(PO_4_)_2._18H_2_O		2.1 (0.1)	3.4 (0.3)	0.1 (0.2)	0.2 (0.2)	0.3 (0.1)	1.8 (0.2)	1.6 (0.3)
Goyazite SrAl_3_P_2_O_7_(OH)_7_	1.5 (0.4)							
7.1 Å-clay^c^	0.8 (0.4)							
10 Å-mica^c^	3.8 (0.6)							
Variscite AlPO_4_.2H_2_O						15.4 (0.4)		
**Total**	100	100	100	100	100	100	100	100

a Goodness-of-fit indicator *R*_*wp*_ for the weighted profile: *R*_*wp*_= [(Σ*w*_*i*_(*y*_*io*_*−y*_*ic*_)^2^)/(Σ*w*_*i*_*y*_*io*_^2^)]^1/2^, where *y*_*io*_ is the observed intensity, *y*_*ic*_ the calculated intensity, and *w*_*i*_ the weight assigned to each observation based on counting statistics.

b Refined variables included zero correction, scale factors, unit cell parameters of major phases and up to four peak shape parameters per mineral.

c 7.1 Å-clay is likely kaolinite and 10 Å-mica is likely illite or muscovite.

**Table 2:** SEM-EDS analyses of the phases and mixed phases in RL32-B-2011.

**Mineral**	Whewellite		Crandallite	Sr crandallite	Crandallite
**SEM analysis of**	>1 phase	>1 phase	1 phase	1 phase	>1 phase
**Analysis #**	#15	#7	#1	#26	#13
**wt% (norm C free)**					
**SiO**_**2**_	6.5	5.22	0	0.2	2.42
**Al**_**2**_**O**_**3**_	5.15	7.09	42.83	37.73	8.17
**FeO**	1.29		1.08	0.27	
**MgO**	0	0	0.01	0	0
**CaO**	79.36	68.48	9.87	11.23	43.65
**SrO**				11.14	
**Na**_**2**_**O**	0.74	1.13	0.57	0	1.23
**K**_**2**_**O**	1.25	3.9	1.41	0.19	2.62
**P**_**2**_**O**_**5**_	5.37	11.06	37.56	29.64	39.51
**SO**_**3**_	0	2.5	6.35	9.52	1.11
**Cl**	0.35	0.62	0.32	0.08	1.29
**Atomic formula unit, based on:**	**4 O + 2 C**	**4 O + 2 C**	**13 O**	**13 O**	**11 O**
**Si**	0.21	0.17	0	0.02	0.17
**Al**	0.2	0.27	3.84	3.8	0.69
**Fe total**	0.04	0	0.07	0.02	0
**Mg**	0	0	0	0	0
**Ca**	1.40*	1.18*	0.8	1.03	3.34
**Sr**				0.55	
**Na**	0.05	0.07	0.08	0	0.17
**K**	0.05	0.16	0.14	0.02	0.24
**P**	0.15	0.3	2.42	2.14	2.39
**Cl**	0.02	0.03	0.04	0.01	0.16
**TOTAL**	1.95	1.84	4.82	5.23	4.59
**Ideal Formula**	CaC_2_O_4_.H_2_O		CaAl_3_(PO_4_)_2_	(Ca,Sr)Al_3_(PO_4_)_2_	Ca_3_Al(PO_4_)_2_
			(OH)_5_.H_2_O	(OH)_5_.H_2_O	(OH)_3_.H_2_O
					
**Measured**			Ca_0.8_Al_3.84_	(Ca,Sr)_1.58_Al_3.8_	
**Formula**			(PO_4_)_2.42_	(PO_4_)_2.14_	
			(OH)_5_.H_2_O	(OH)_5_.H_2_O	

**Table 3:** Sample identification and location.

Sample	Latitude	Longitude
RLL032-B-2011	12°23׳49.55"S	133° 0׳26.81"E
RLL032-B-S1	12°23׳49.55"S	133° 0׳26.81"E
RLL032-B-S2	12°23׳49.55"S	133° 0׳26.81"E
RLL032-B-S3	12°23׳49.55"S	133° 0׳26.81"E
RLL032-B-S4	12°23׳49.55"S	133° 0׳26.81"E
RLL3-1-1	12°24׳7.59"S	133° 0׳5.51"E
RLL3-1-2	12°24׳7.59"S	133° 0׳5.51"E
RLL3-1-3	12°24׳7.59"S	133° 0׳5.51"E

## Appendix A Supplementary material

Supplementary material **Supplementary Figure 1** (SuppFigure1_Mineral_ID_XRD_scans.docx). Specific minerals fit to X-ray Diffraction data for the samples. **Supplementary Figure 2** (SuppFigure2_Mineral_Quant_Rietveld. docx). Rietveld refinement results of minerals fit to X-ray Diffraction data for the samples, plus residuals. **Supplementary Table 1** (SuppTable1_BulkSamples_XRD_Raw_Data.xlsx). Raw X-ray Diffraction data for the samples. **Supplementary Table 2** (SuppTable2_FTIR_Band_Assignment.docx). Relevant infrared bands identified in the literature. **Supplementary Table 3** (SuppTable3_BulkSamples_FTIR_Raw_Data.xlsx). Raw Fourier Transform Infrared data for the samples.
