# Supplementary material for: Characterization of mineral coatings associated with a Pleistocene‐Holocene rock art style: The Northern Running Figures of the East Alligator River region, western Arnhem Land, Australia
Source: Data Brief. 2016 Dec 20;10:537–43. doi: 10.1016/j.dib.2016.12.024 (PMC5219645; doi:10.1016/j.dib.2016.12.024)
Supplement: Supplementary file 2 — Supplementary material Supplementary Figure 1 (SuppFigure1_Mineral_ID_XRD_scans.docx). Specific minerals fit to X-ray Diffraction data for the samples. Supplementary Figure 2 (SuppFigure2_Mineral_Quant_Rietveld. docx). Rietveld refinement results of minerals fit to X-ray Diffraction data for the samples, plus residuals. Supplementary Table 1 (SuppTable1_BulkSamples_XRD_Raw_Data.xlsx). Raw X-ray Diffraction data for the samples. Supplementary Table 2 (SuppTable2_FTIR_Band_Assignment.docx). Relevant infrared bands identified in the literature. Supplementary Table 3 (SuppTable3_BulkSamples_FTIR_Raw_Data.xlsx). Raw Fourier Transform Infrared data for the samples. [file mmc2.zip › SuppFigure2_Mineral_Quant_Rietveld.docx]

The following plots show the raw XRD data in black crosses and the Rietveld refinement fit in red. The residual between the data and the calculated Rietveld refinement fit is shown in green. Areas omitted from the Rietveld analysis routine are shown in light blue. Individual peak positions are given in medium blue (as indicated in the tables).
