# Supplementary material for: Characterization of mineral coatings associated with a Pleistocene‐Holocene rock art style: The Northern Running Figures of the East Alligator River region, western Arnhem Land, Australia
Source: Data Brief. 2016 Dec 20;10:537–43. doi: 10.1016/j.dib.2016.12.024 (PMC5219645; doi:10.1016/j.dib.2016.12.024)
Supplement: Supplementary file 2 — Supplementary material Supplementary Figure 1 (SuppFigure1_Mineral_ID_XRD_scans.docx). Specific minerals fit to X-ray Diffraction data for the samples. Supplementary Figure 2 (SuppFigure2_Mineral_Quant_Rietveld. docx). Rietveld refinement results of minerals fit to X-ray Diffraction data for the samples, plus residuals. Supplementary Table 1 (SuppTable1_BulkSamples_XRD_Raw_Data.xlsx). Raw X-ray Diffraction data for the samples. Supplementary Table 2 (SuppTable2_FTIR_Band_Assignment.docx). Relevant infrared bands identified in the literature. Supplementary Table 3 (SuppTable3_BulkSamples_FTIR_Raw_Data.xlsx). Raw Fourier Transform Infrared data for the samples. [file mmc2.zip › SuppTable2_FTIR_Band_Assignment.docx]

Supplementary Table 2: FTIR band assignments. Bands are shown in Figure 1a, b.

| **IR band (cm^-1^)** | **Molecular Group**  **(& Mineral)** | **Reference** |
| --- | --- | --- |
| 3486 | OH (phosphate or sulfate) | [7] |
| 3420 | OH (oxalate) | [8] |
| 3344 | OH | [7] |
| 3246 | Al_2_-OH | [9] |
| 3062 | OH | [7] |
| 1625 | HOH bending (oxalate, gypsum) | [7, 8] |
| 1430 | CO_3_ or SO_4_ (gypsum) or C_2_O_4_ (whewellite) or (NH_4_)^+^ | [7, 8] |
| 1383 | PO_4_ | [7] |
| 1315-20 | C_2_O_4_ (oxalate) | [8] |
| 1108-12 | PO_4_ (phosphate) | [7] |
| 1020 | PO_4_ (phosphate) or SiO_4_ (muscovite or clay) | [7, 10] |
| 890 | PO_4_ (phosphate) | [7, 10] |
| 780 | C_2_O_4_ (oxalate) or SiO_4_ (quartz) | [7] |
| 670 | C_2_O_4_ (oxalate) or SO_4_ (gypsum) or CO_3_ (carbonate) or PO_4_ (phosphate) | [7, 8] |
